# Salt stress induces changes in the proteomic profile of micropropagated sugarcane shoots

**DOI:** 10.1371/journal.pone.0176076

**Published:** 2017-04-18

**Authors:** Lucas Z. Passamani, Roberta R. Barbosa, Ricardo S. Reis, Angelo S. Heringer, Patricia L. Rangel, Claudete Santa-Catarina, Clícia Grativol, Carlos F. M. Veiga, Gonçalo A. Souza-Filho, Vanildo Silveira

**Affiliations:** 1 Laboratório de Biotecnologia, Centro de Biociências e Biotecnologia (CBB), Universidade Estadual do Norte Fluminense Darcy Ribeiro (UENF), Campos dos Goytacazes, RJ, Brazil; 2 Unidade de Biologia Integrativa, Setor de Genômica e Proteômica, UENF, Campos dos Goytacazes, RJ, Brazil; 3 Laboratório de Biologia Celular e Tecidual, CBB, UENF, Campos dos Goytacazes, RJ, Brazil; 4 Laboratório de Química e Função de Proteínas e Peptídeos, CBB, UENF, Campos dos Goytacazes, RJ, Brazil; 5 Laboratório de Cultura de Tecidos Vegetais (Biofábrica), Universidade Federal Rural do Rio de Janeiro Campus Campos dos Goytacazes, Campos dos Goytacazes, RJ, Brazil; Louisiana State University College of Agriculture, UNITED STATES

## Abstract

Salt stress is one of the most common stresses in agricultural regions worldwide. In particular, sugarcane is affected by salt stress conditions, and no sugarcane cultivar presently show high productivity accompanied by a tolerance to salt stress. Proteomic analysis allows elucidation of the important pathways involved in responses to various abiotic stresses at the biochemical and molecular levels. Thus, this study aimed to analyse the proteomic effects of salt stress in micropropagated shoots of two sugarcane cultivars (CB38-22 and RB855536) using a label-free proteomic approach. The mass spectrometry proteomics data are available via ProteomeXchange with identifier PXD006075. The RB855536 cultivar is more tolerant to salt stress than CB38-22. A quantitative label-free shotgun proteomic analysis identified 1172 non-redundant proteins, and 1160 of these were observed in both cultivars in the presence or absence of NaCl. Compared with CB38-22, the RB855536 cultivar showed a greater abundance of proteins involved in non-enzymatic antioxidant mechanisms, ion transport, and photosynthesis. Some proteins, such as calcium-dependent protein kinase, photosystem I, phospholipase D, and glyceraldehyde-3-phosphate dehydrogenase, were more abundant in the RB855536 cultivar under salt stress. Our results provide new insights into the response of sugarcane to salt stress, and the changes in the abundance of these proteins might be important for the acquisition of ionic and osmotic homeostasis during exposure to salt stress.

## Introduction

Sugarcane (*Saccharum* spp.) is an important crop in several countries and Brazil is the largest producer of sugarcane in the world [1]. The expansion of sugarcane cultivation is economically important for the commercialization of its main products, sugar, and ethanol [2]. Although it is well-suited to high temperature, sugarcane is adversely affected by other abiotic stresses, such as drought and salt stress [3]. In fact, salt stress constitutes one of the best-studied stresses due to concerns regarding continuing increases in soil salinity. More than 50% of all arable land will have high concentrations of salt in 2050 [4]. Proximity to the ocean favours the accumulation of salt in coastal soils; however, other regions can also have saline soils depending on the water used for irrigation, which usually has a high NaCl content and precipitation levels [5].

Salinity affects the productivity of various plant species [6,7] and can cause morphological, physiological, biochemical, and molecular changes [8]. Previous studies have suggested that the effects of salt stress are multifactorial; in particular, a high salt concentration causes osmotic stress and consequent ionic stress, primarily due to high concentrations of Na^+^ [9]. Osmotic stress decreases water absorption by the roots and causes various physiological effects, such as low photosynthetic activity and the production of reactive oxygen species (ROS) [7]. Ionic stress is caused by an increased absorption of ions, primarily Na^+^ and Cl^-^, which results in biochemical and physiological damage. In particular, high concentrations of intracellular Na^+^ prevent the uptake of K^+^, which is an essential element in several cellular processes [10].

According to Zhu [11], salt tolerance depends on the interconnections among the biochemical pathways involved in detoxification, homeostasis, and growth regulation. First, accumulated ROS are removed through the synthesis of important compounds, such as osmolytes and several proteins that decrease the intracellular concentration of ROS. Simultaneously, ionic homeostasis is achieved via the compartmentalization of ions in vacuoles. Finally, the expression of important genes is regulated to efficiently maintain plant growth and high productivity [11].

Salt tolerance is thus believed to be a complex process involving physiological, biochemical, and molecular responses [12]. Proteomic analysis is fundamentally important for understanding the mechanisms of the responses of plants because these analyses provide important information regarding the dynamics of protein expression and post-translational modifications involved in stress-related events [13]. Advances in protein identification techniques have made it possible to identify proteins involved in the tolerance of various species to abiotic stresses [14–16]. Recent studies on crop plants have identified the following five main groups of proteins that present differential abundance and are directly related to salt tolerance mechanisms in plants: I) heat shock proteins (HSPs), II) late embryogenesis abundant proteins (LEA proteins), III) osmolyte biosynthetic enzymes, IV) proteins involved in carbon metabolism, and V) enzyme scavengers of ROS [17]. In addition to these groups, proteins associated with ion transport, protein synthesis/degradation, and signal transduction are also important in response to salt stress in major crops, such as rice (*Oryza sativa*) [18], wheat (*Triticum durum* L.) [16], and sugar beet (*Beta vulgaris* L.) [19]. Capriotti et al. [16] suggested that the proteins identified in these studies might constitute useful molecular markers for future breeding programmes.

In recent years, the available knowledge regarding the mechanisms underlying the tolerance of sugarcane to salt stress has increased. These recent studies have identified genes and metabolic pathways that might be important in the response to salt stress; in particular, proteins associated with carbohydrate metabolism and energy might be involved in the response of sugarcane to salt stress [20,21]. Although these studies have increased our understanding of these events, the biochemical and molecular changes that occur in different varieties of sugarcane remain relatively unknown. Understanding the plant´s response to salt stress is difficult because the morphological characteristics of plants, which present differences among species, particularly among the root systems of different species, represent an important aspect of the tolerance mechanisms. Thus, the combination of molecular and morphological differences between sensitive and tolerant cultivars makes it difficult to determine the specific role of each tolerance factor. In this context, the combination of proteomic analysis with tissue culture, which minimizes varietal differences in the root architecture and provides a controlled environment, will aid the elucidation of the main metabolic pathways that are regulated in sugarcane under salt stress.

Although it is known that sugarcane is strongly affected by salt stress, knowledge regarding the cellular, biochemical, and molecular mechanisms of the response of sugarcane to salt stress is lacking. Thus, proteomic analysis conducted in this study, might help the identification and understanding of novel response mechanisms of sugarcane to salt stress. In addition, the information obtained through differential protein analysis might be used in modern breeding programmes for the production of new cultivars [22]. Therefore, this work aimed to evaluate changes in the proteome of micropropagated shoots of two sugarcane cultivars (CB38-22 and RB855536) under salt stress using a label-free proteomic approach to better understand the mechanisms controlling the responses of sugarcane plants to salt stress.

## Materials and methods

### Plant materials and growth conditions

Sugarcane plants (cv. CB38-22 and cv. RB855536) were obtained from the Universidade Federal Rural do Rio de Janeiro (UFRRJ), Campus Campos dos Goytacazes, located in Campos dos Goytacazes, RJ, Brazil (21°48’S and 41°17’W). Both cultivars have a high sucrose content and exhibit a high agroindustrial productivity. CB38-22 is a cultivar with a curved posture that can be planted in soils with average to high fertility [23]. Farmers consider CB38-22 tolerant to salt stress, although no conclusive experimental evidence exists. RB855536 is characterized by a more erect posture and resistance to brown rust, leaf scald, mosaic, and coal viruses. Its planting in favourable environments is recommended due to its low tolerance to water stress [24]. Water stress is one of the main factors that affect sugarcane productivity [25], and its effects are very similar to those of salt stress. Thus, these cultivars were selected for use in this study because the available knowledge regarding their molecular responses to salt stress is scarce, and increased knowledge of these cultivars will be important for sugarcane cultivation.

Four-week-old plants that were cultivated in a field trial were used as sources of explants for *in vitro* culture. After removing the mature leaves, the remaining leaf roll was surface-sterilized with 70% ethanol for 1 min and with 1.25% sodium hypochlorite (v/v) for 20 min. Some leaf primordia were then excised to obtain a cylinder of approximately 0.5 x 5 cm, and the material was washed in sterile distilled water and transferred to a laminar flow cabinet. In the laminar flow cabinet, the surface of the cylinder was disinfected with 1.25% sodium hypochlorite solution for 20 min and subjected to three washes with sterile water. The shoot tips were excised near the meristems and inoculated in liquid MS culture medium [26] (PhytoTechnology Laboratories, Overland Park, KS, USA) supplemented with 2% sucrose, 0.887 μM benzylaminopurine (BA, Sigma-Aldrich, St. Louis, MO, USA), and 0.46 μM kinetin (KIN, Sigma-Aldrich), and the pH of the medium was adjusted to 5.8. The medium was dispensed into test tubes (150 x 25 mm) containing 10 mL of MS medium per tube before autoclaving at 121°C and 152 kPa for 15 min. After inoculation, the explants were incubated for seven days at 25°C in the absence of light. Subsequently, the first leaf primordia were multiplied in periods of 15 days in the same MS medium and incubated in a growth chamber at 20 ± 3°C with a photoperiod of 16 h (38 μmol/m^2^/s).

### Induction of salt stress

To induce salt stress, the sugarcane cultivars CB38-22 and RB855536 were incubated in liquid MS culture medium supplemented with 2% sucrose, 0.887 μM BA, 0.46 μM KIN, and 0 mM (control) or 180 mM NaCl. The NaCl concentration of 180 mM was selected by evaluating a salt stress curve that was generated to determine the optimal NaCl concentration for inducing salt stress (S1 Fig). The pH of the medium was adjusted to 5.8, and the medium was dispensed into culture flasks with filters that allow gas exchange (Bio-sama) (95 x 60 mm). Each flask contained 30 mL of MS medium and was autoclaved at 121°C and 152 kPa for 15 min. The plantlets were distributed randomly among the different culture flasks and maintained for 20 days in a growth chamber at 23 ± 2°C with a photoperiod of 16 h (38 μmol/m^2^/s). After 20 days of exposure to the different treatments, the shoots were collected and stored at -20°C for proteomic analysis.

The experiments were performed using a randomized factorial design consisting of a 2x2 factorial arrangement with five replicates. Each experimental sample consisted of a flask containing one cluster of shoots.

### Proteomic analysis

#### Extraction and quantification of proteins

Proteins were extracted using the method described by Damerval et al. [27]. Five biological replicates (500 mg of fresh matter each) were homogenized in liquid nitrogen using a mortar and pestle. Each biological replicate consisted of one flask containing one plantlet. The resulting powder was placed in a 2-mL microtube, and 1 mL of extraction buffer [10% trichloroacetic acid (TCA; Sigma-Aldrich) in acetone] was added. The samples were incubated for 60 min at 4°C and then centrifuged at 16,000 g for 30 min. The supernatant was discarded, and the pellet was washed three times in cold acetone with 20 mM dithiothreitol (DTT; Bio-Rad Laboratories, Hercules, CA, USA). Subsequently, the pellets were resuspended in 1 mL of buffer containing 7 M urea (GE Healthcare, Piscataway, NJ, USA), 2 M thiourea (GE Healthcare), 1% DTT (Bio-Rad Laboratories), 2% Triton-100 (GE Healthcare), and 1 mM phenylmethanesulfonyl fluoride (PMSF; Sigma-Aldrich) and stirred for 60 min at 4°C until the samples were completely resuspended. The samples were incubated on ice for 30 min and then centrifuged at 16,000 g for 10 min. The supernatants were then collected and stored at -20°C. The total protein concentration was determined using a 2-D Quant Kit (GE Healthcare).

#### Protein digestion

Total protein samples (100 μg) were prepared according to Reis et al. [28]. Before trypsin digestion, the samples were desalted on 5000 MWCO Vivaspin 500 membranes (GE Healthcare) using 50 mM ammonium bicarbonate (pH 8.5; Sigma-Aldrich) as the buffer. The membranes were loaded to their maximum capacity with ammonium bicarbonate buffer and centrifuged at 15,000 g and 8°C for 20 min. This procedure was repeated at least three times until approximately 50 μL of the sample remained.

The methodology used for protein digestion was previously described by Heringer et al. [29]. Twenty-five microliters of 0.2% (v/v) RapiGest® surfactant (Waters, Milford, CT, USA) was added to each sample. The resulting mixtures were briefly vortexed and incubated in an Eppendorf Thermomixer® for 15 min at 80°C, and 2.5 μL of 100 mM DTT was added. After the tubes were vortexed and incubated at 60°C for 30 min with agitation, 2.5 μL of 300 mM iodoacetamide (GE Healthcare) was added, and the samples were vortexed and incubated in the dark for 30 min at room temperature. Digestion was performed by adding 20 μL of trypsin solution (50 ng/μL; V5111, Promega, Madison, WI, USA) prepared in 50 mM ammonium bicarbonate and then incubating the samples overnight at 37°C. For RapiGest® precipitation, 10 μL of 5% (v/v) trifluoroacetic acid (TFA, Sigma-Aldrich) was added, and the samples were incubated at 37°C for 90 min and then centrifuged at 16,000 g for 30 min. The samples were subsequently transferred to Total Recovery Vials (Waters) for mass spectrometry analysis.

#### Mass spectrometry analysis

A nanoAcquity UPLC connected to a Synapt G2-Si HDMS mass spectrometer (Waters) was used for ESI-LC-MS/MS analysis according to Reis et al. [28]. Chromatography was performed by injecting 1 μL of each digested sample for normalization prior to the relative quantification of proteins. To ensure standardized molar values for all conditions, the normalization among samples was based on stoichiometric measurements of the total ion counts of scouting runs performed prior to the analyses. After normalization, the injection volumes were adjusted to ensure the injection of equal protein quantities for each sample. The runs consisted of three technical replicates. During separation, the samples were loaded onto the nanoAcquity UPLC 5-μm C18 trap column (180 μm x 20 mm) at 5 μL/min for 3 min and then onto the nanoAcquity HSS T3 1.8-μm analytical reverse-phase column (100 μm x 100 mm) at 600 nL/min. The column temperature was 60°C. For peptide elution, a binary gradient was used: mobile phase A consisted of water (Tedia, Fairfield, Ohio, USA) and 0.1% formic acid (Sigma-Aldrich), and mobile phase B consisted of acetonitrile (Sigma-Aldrich) and 0.1% formic acid. Gradient elution was performed as follows: 7% B for 3 min, ramping from 7 to 40% B until 90.09 min, ramping from 40 to 85% B until 94.09 min, holding constant at 85% until 98.09 min, decreasing to 7% B until 100.09 min, and holding constant at 7% B until the end of the run at 108.09 min. Mass spectrometry was performed in the positive and resolution mode (V mode) with a resolution of 35000 FWHM with ion mobility and in the data-independent acquisition mode. The IMS wave velocity was set to 600 m/s; the transfer collision energy was ramped from 19 V to 45 V in the high-energy mode; the cone and capillary voltages were 30 V and 2800 V, respectively; and the source temperature was 70°C. The nano flow gas was set to 0.50 Bar, and the purge gas flow ranged from 145 to 150 L/h. The TOF parameters included a scan time of 0.5 s in the continuum mode and a mass range of 50 to 2000 Da. Human [Glu1]-fibrinopeptide B (Sigma-Aldrich) at 100 fmol/μL was used as an external calibrant, and lock mass acquisition was performed every 30 s.

#### Bioinformatics analysis

The spectra processing and database searching conditions were established using Progenesis QI for Proteomics Software v.2.0 (Nonlinear Dynamics, Newcastle, UK). The Progenesis software platforms use the Apex3D algorithm (Waters Corporation), which processes the data using a low-energy threshold of 135 (counts), an elevated energy threshold of 30, and an intensity threshold of 750. In addition, the analysis was performed using the following parameters: one missed cleavage, minimum fragment ion per peptide equal to 1, minimum fragment ion per protein equal to 3, minimum peptide per protein equal to 1, fixed modifications of carbamidomethyl and variable modifications of oxidation and phosphoryl, a default maximum false discovery rate of 4%, a score greater than 5, and a maximum mass error of 10 ppm. The Sorghum bicolour protein sequence set was downloaded from JGI (ftp://ftp.jgi-psf.org/pub/compgen/phytozome/v9.0/Sbicolor/annotation/Sbicolor79protein.fa.gz). This file was used as a reference database for peptide mapping using Progenesis software. For the comparison of protein abundance among different samples, each run was defined as an individual condition to enable the reporting of protein quantities as single values for subsequent data analysis. After Progenesis analysis, only the proteins present in all three runs with a coefficient of variation less than 0.5 were included to ensure the quality of the results. The proteins that presented differential abundances were considered to be up-regulated if the log_2_ of the fold change (FC) was greater than 0.5 and down-regulated if the log_2_ of the FC was less than -0.5 (both P<0.05). Using MapMan software (v. 3.6.0RC1) and Blast2GO (v. 3.1), functional annotation of the proteins that presented differential abundances among the samples subjected to the different treatments was performed. The mass spectrometry proteomics data have been deposited to the ProteomeXchange Consortium [30] via the PRIDE [31] partner repository with the dataset identifier PXD006075".

## Results

**Fig 1:**
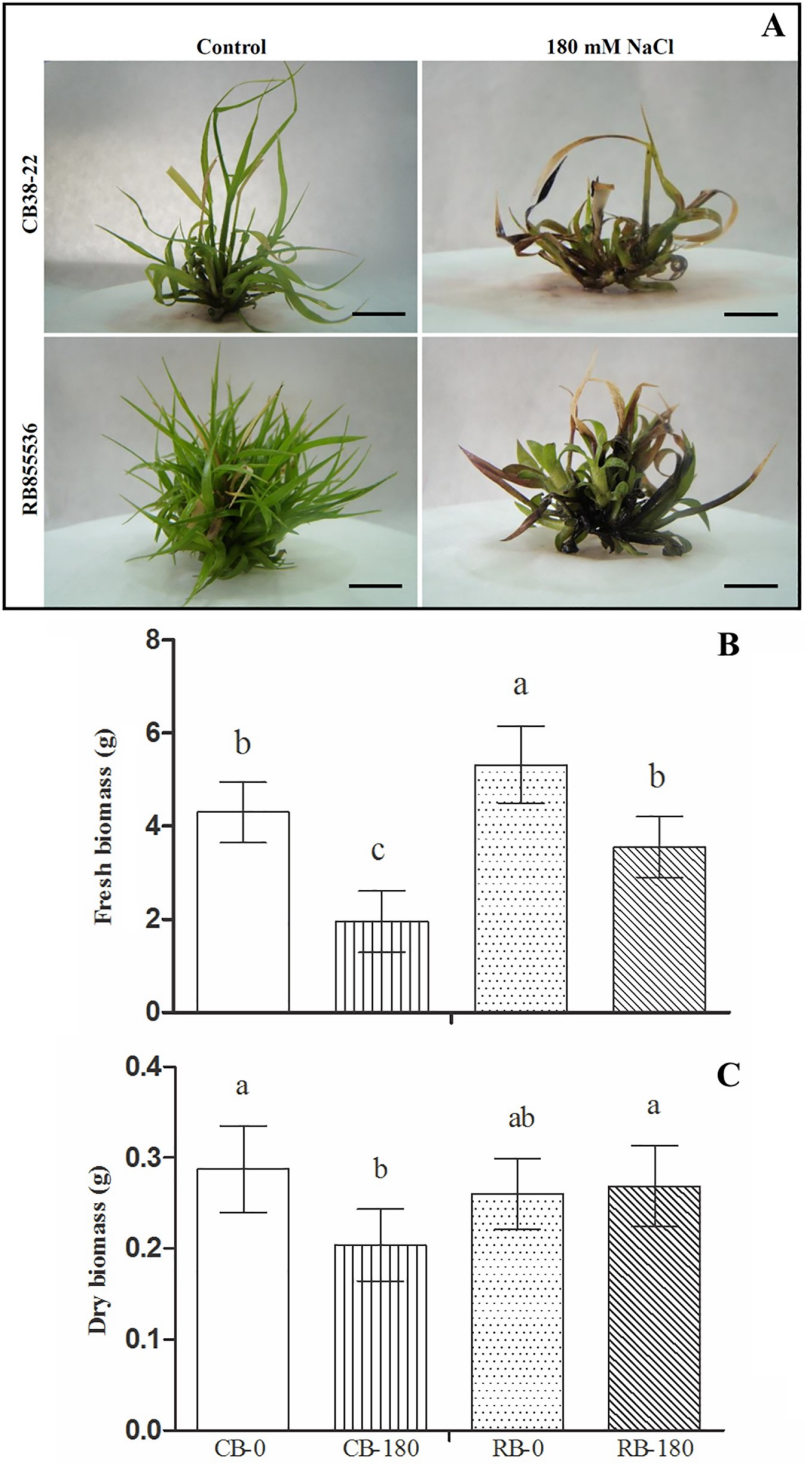
**Morphological characteristics (A), fresh matter (B) and dry matter (C) of micropropagated plantlets of the sugarcane cultivars CB38-22 and RB855536 in liquid MS culture media with 0 mM NaCl (control) or 180 mM NaCl.** Bars = 0.75 cm. The same letter above the bars indicates no significant difference according to Tukey’s test at 5% probability.

To analyse the effects of salinity on the developmental characteristics of RB855536 and CB38-22, *in vitro* shoots of the two cultivars were exposed to 0 mM (control) of 180 mM NaCl for 20 days and were evaluated in terms of fresh and dry matter. The salt stress treatment caused significant reductions in the fresh matter of RB855536 and CB38-22 compared with the control plants (Fig 1). Notably, CB38-22 presented a greater reduction in its aerial growth, indicating that this cultivar is more affected by salt stress (Fig 1).

The comparison of the two cultivars showed that CB38-22 presented a more pronounced growth reduction than RB855536 (55% vs. 33%). The response of each cultivar to exposure to 180 mM NaCl showed differences in the dry matter parameter: the RB855536 cultivar did not change in comparison to its control, and the CB38-22 cultivar showed a reduction of 29%, confirming its greater sensitivity to salt stress.

Based on the proteomic analysis, we identified 1172 proteins and 1160 of these were observed in both cultivars in the absence or presence of NaCl (CB-0, CB-180, RB-0, and RB-180 treatments; S2 Fig). Seven proteins were shared specifically among the CB-180, RB-0, and RB-180 treatments; one protein was observed in both the CB-0 and RB-180 treatments; one protein was shared specifically between the RB-0 and RB-180 treatments; and three proteins were observed in both cultivars specifically under salt stress (CB-180 and RB-180 treatments). The three proteins shared by the two cultivars under salt stress were MEMO1-like (Sb03g046030.1), hexokinase 1 (Sb09g026080.1), and glutathione S-transferase (GST; Sb03g044550.2). A complete list of all the proteins identified in the cultivars subjected to the different treatments is presented in S1 Table.

**Fig 2:**
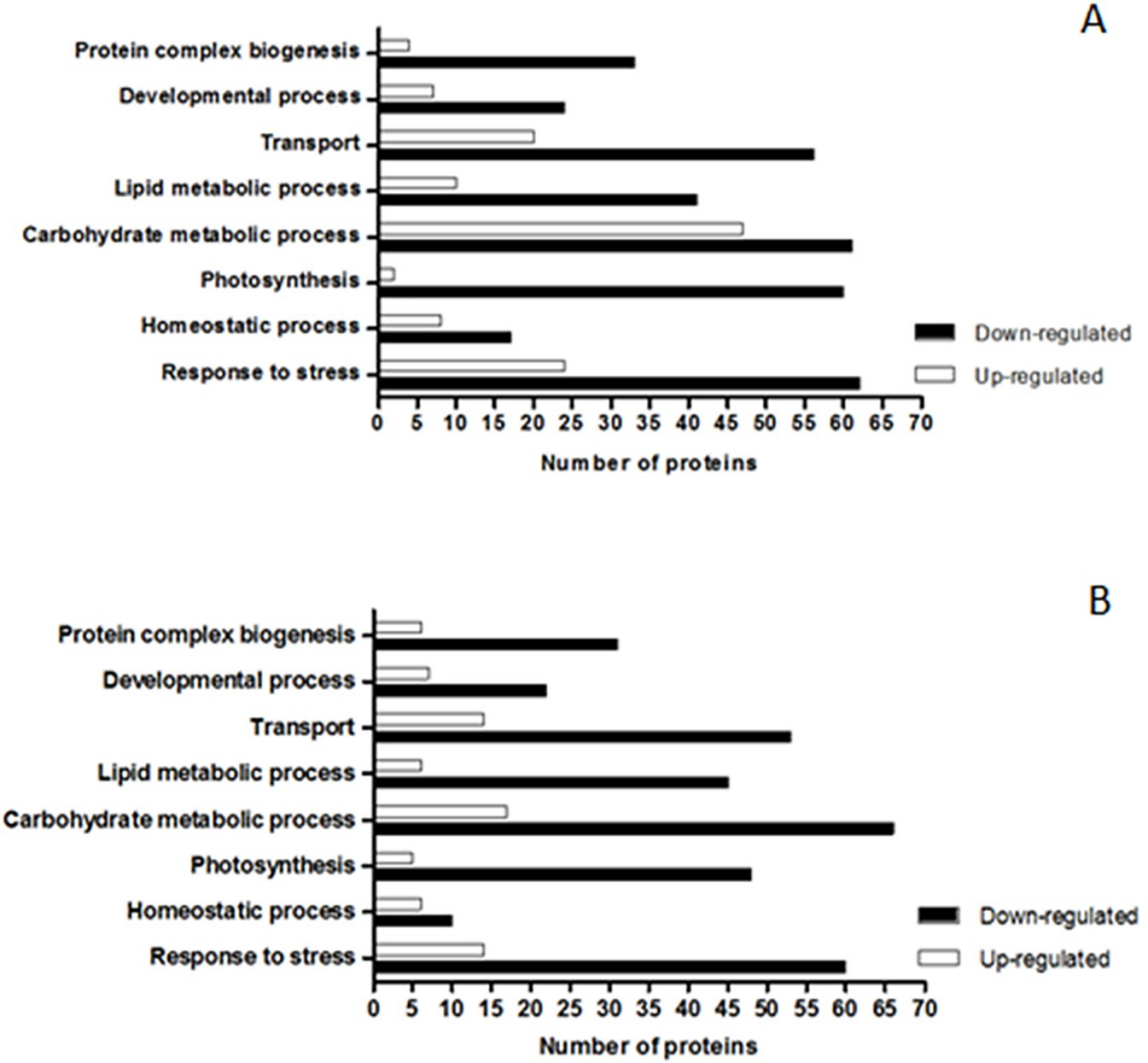
Functional analysis of proteins showing differential abundance between cultivars CB38-22 and RB855536 under salt stress (180 mM NaCl) compared with their respective controls (without NaCl). A = CB-180/CB-0. B = RB-180/RB-0. The log_2_ values of the fold changes obtained for each cultivar under salt stress compared with their respective controls were used to determine the levels of protein abundance. The up-regulated proteins showed log_2_ values greater than 0.5, and the down-regulated proteins showed log_2_ values less than -0.5.

Among the 1160 proteins observed under all conditions, the majority showed similar patterns of abundance in both varieties under salt stress. In the CB38-22 cultivar, 420, 506, and 235 proteins presented lower, unchanged, and higher abundance under salt stress compared with the control, respectively. In contrast, in the RB855536 cultivar, 468, 560, and 140 proteins exhibited lower, unchanged, and higher abundance compared with the control, respectively. A GO analysis of the proteins presenting differential abundances proteins, which aimed to identify the main biological processes regulated by salt stress in each cultivar, was performed using the Blast2GO programme (Fig 2). The analysis revealed that the majority of proteins regulated by salt stress in both cultivars are involved in the processes of carbohydrate metabolism, response to stress, transport, and photosynthesis. The comparison of the two cultivars under salt stress revealed that the abundance of proteins related to photosynthesis and protein complex biogenesis is higher in the RB855536 cultivar. In contrast, proteins related to the processes of carbohydrate metabolism, lipid metabolism, homeostasis, transport, and response to stress presented increased abundance in the CB38-22 cultivar.

**Fig 3:**
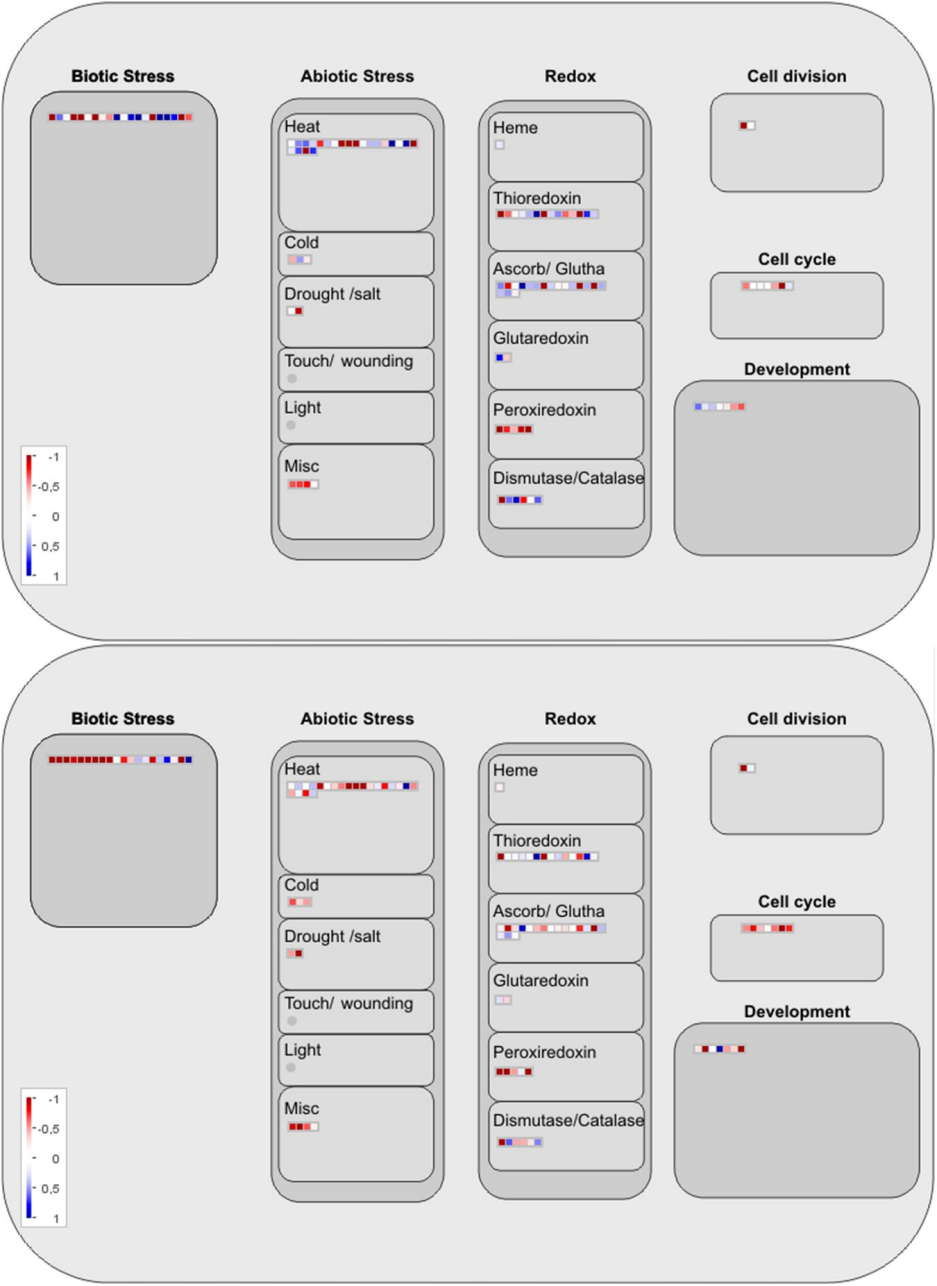
Protein mapping of the CB38-22 and RB855536 cultivars under salt stress (180 mM NaCl) compared with their respective controls (without NaCl). A = CB-180/CB-0. B = RB-180/RB-0. All proteins with functions related to “response to biotic and abiotic stresses” are displayed on the maps. The log_2_ values of the fold changes obtained for each cultivar under salt stress compared to their respective controls were used to determine the levels of protein abundance.

The pattern reflecting the regulation of proteins with differential abundances in the two cultivars in response to saline stress was analysed using MapMan software (Fig 3). An analysis of the major cellular response categories revealed that a greater number of these proteins in CB38-22 were involved in the response to abiotic stress and the redox response. In the RB855536, a greater number of the down-regulated proteins were found to be involved in the antioxidant response (Fig 3).

**Fig 4:**
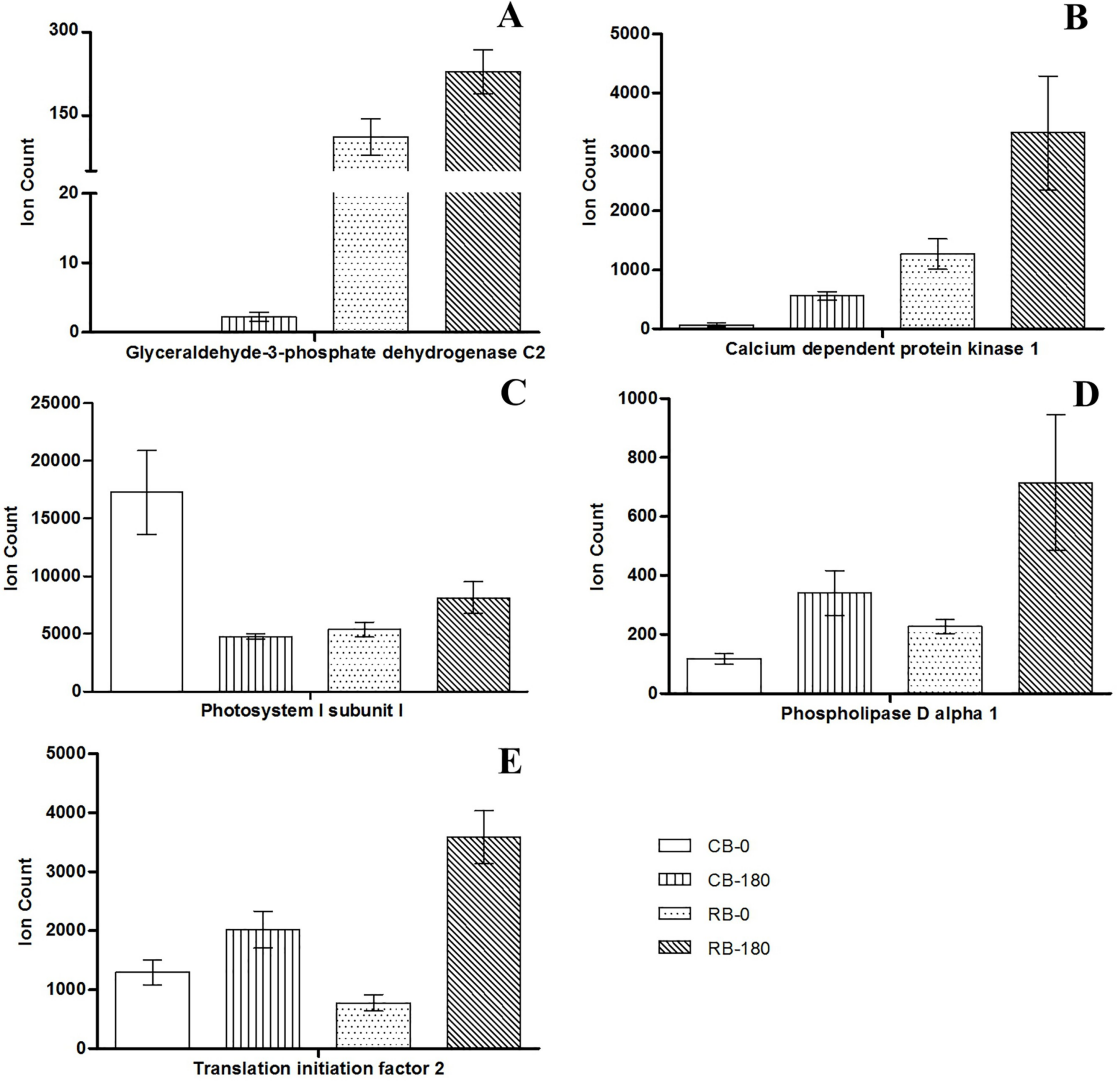
Differential abundance of proteins with important roles in the response of sugarcane plants to salt stress. The abundances of glyceraldehyde-3-phosphate dehydrogenase (A), calcium-dependent protein kinase 1 (B), photosystem I subunit I (C), phospholipase D alpha 1 (D), and translation initiation factor 2 (E) are shown for both cultivars under control and salt stress conditions. The bars represent the means ± SD (n = 3). CB-0 and RB-0 = cultivar CB38-22 and RB855536, respectively, cultured without NaCl (controls); CB-180 and RB-180 = cultivars CB38-22 and RB855536, respectively, cultured with 180 mM NaCl.

Differences in protein abundance might be a common response to salt stress or might reveal genotype-specific alterations. The comparison of dissimilar cultivars can reveal both common and genotype-dependent responses. The analysis of the abundance patterns of proteins that show significant alteration in both varieties but present differences in abundance between tolerant and sensitive genotypes might provide more accurate conclusions regarding the biological roles of these proteins during exposure to salinity stress. Twenty-three proteins with differential abundance between the two cultivars were found and these might reflect potential genotype-specific responses of sugarcane to salt stress (Table 1). Proteins such as photosystem I subunit l (Sb03g004560.1), glyceraldehyde-3-phosphate dehydrogenase (GAPC; Sb09g001600.1), calcium-dependent protein kinase (CDPK; Sb08g014910.1), phospholipase D (PLD; Sb10g023630.1), and translation initiation factor 2 (eIF2; Sb06g017820.1) were differentially regulated under salt stress (Fig 4). These proteins are known to exert direct and indirect effects on the responses to various abiotic stresses and might therefore be important proteins involved in the response of sugarcane to salt stress (Fig 4 and Table 1).

**Table 1:** Proteins that were up-regulated or down-regulated under salt stress compared with their respective controls (CB-180/CB-0; RB-180/RB-0*) and proteins showing differential regulation between the two cultivars (RB-180/CB-180).

Accession	Peptide Count	Score	Log_2__FC			Relative abundance regulation			Description	Function
			CB-180/ CB-0	RB-180/ RB-0	RB-180/ CB-180	CB-180/ CB-0	RB-180/ RB-0	RB-180/ CB-180		
Sb03g004560.1	2	15.1	-1.859	0.599	0.774	DOWN	UP	UP	Photosystem I subunit l	Photosynthesis
Sb02g006860.1	2	11.9	-1.229	0.648	1.876	DOWN	UP	UP	Galactose oxidase/kelch repeat superfamily protein	Lipid transport
Sb04g023210.1	1	6.0	0.076	0.871	0.810	UNCHANGED	UP	UP	Glutathione S-transferase (class zeta) 2	Response to toxic substance
Sb03g020160.1	3	24.7	-0.066	0.941	1.190	UNCHANGED	UP	UP	Photosystem II reaction centre protein B	Photosynthesis
Sb03g028050.1	12	77.9	-0.397	0.942	0.552	UNCHANGED	UP	UP	Oxidoreductase, aldo/keto reductase family protein, putative, expressed	Starch biosynthetic process, maltose metabolic process
Sb07g002070.1	3	18.0	0.414	1.008	0.804	UNCHANGED	UP	UP	Succinate dehydrogenase 2–2	Mitochondrial electron transport
Sb06g028330.1	8	50.9	Unique CB-180	0.767	0.614	Unique CB-180	UP	UP	Ribosomal protein S11-beta	Translation
Sb09g001600.1	13	144.5	Unique CB-180	1.044	6.657	Unique CB-180	UP	UP	Glyceraldehyde-3-phosphate dehydrogenase C2	Glycolytic process, carbohydrate metabolic process
Sb03g011460.1	2	10.5	Unique CB-180	1.395	0.828	Unique CB-180	UP	UP	Coatomer, beta subunit	Intracellular protein transport
Sb03g025720.1	1	5.6	Unique CB-180	2.106	2.665	Unique CB-180	UP	UP	Carbamoyl phosphate synthetase B	Urea cycle, arginine biosynthetic process
Sb05g004860.1	1	7.1	Unique CB-180	4.399	2.656	Unique CB-180	UP	UP	Cleavage stimulating factor 64	Regulation of gene silencing, gene silencing by RNA
Sb01g037950.1	3	18.7	1.897	0.823	1.157	UP	UP	UP	Pathogenesis-related Bet v I family protein, putative, expressed	Defence response, response to biotic stimulus
Sb02g042550.1	3	18.1	0.777	0.928	0.775	UP	UP	UP	Rubber elongation factor protein (REF)	Translation elongation factor activity
Sb03g028020.1	6	36.7	0.514	0.936	0.586	UP	UP	UP	Oxidoreductase, aldo/keto reductase family protein, putative, expressed	Starch biosynthetic process, maltose metabolic process
Sb03g000850.1	2	22.4	0.847	1.031	0.720	UP	UP	UP	Phosphorylase superfamily protein	Nucleoside metabolic process
Sb09g030530.1	1	5.7	3.083	1.266	0.845	UP	UP	UP	Hypersensitive-induced response protein, putative, expressed	Cellular cation homeostasis, regulation of plant-type hypersensitive response
Sb08g014910.1	2	13.0	3.116	1.389	2.578	UP	UP	UP	Calcium-dependent protein kinase 1	Protein phosphorylation, intracellular signal transduction, abscisic acid-activated signalling pathway
Sb03g032760.1	3	17.9	1.435	0.661	2.140	UP	UP	UP	Myosin family protein with the Dil domain	Actin filament-based movement
Sb09g004430.1	2	10.3	1.318	1.473	1.687	UP	UP	UP	Dihydrolipoyl dehydrogenase, putative, expressed	Cell redox homeostasis, response to cadmium ion
Sb10g023630.1	6	34.7	1.543	1.655	1.071	UP	UP	UP	Phospholipase D alpha 1	Lipid catabolic process, response to abscisic acid, regulation of stomatal movement
Sb04g029040.2	5	29.8	0.526	1.663	1.065	UP	UP	UP	Aldehyde dehydrogenase 2B7	Aldehyde dehydrogenase (NAD) activity
Sb04g027630.1	3	26.2	4.756	2.095	2.025	UP	UP	UP	Lipase/lipoxygenase, PLAT/LH2 family protein	Jasmonic acid biosynthetic process, response to wounding
Sb06g017820.1	1	5.6	0.645	2.209	0.829	UP	UP	UP	Translation initiation factor 2, small GTP-binding protein	Protein biosynthesis, initiation factor

*CB-0 and RB0 = cultivars CB38-22 and RB855536, respectively, cultured without NaCl (control); CB-180 and RB-180 = cultivars CB38-22 and RB855536, respectively, cultured with 180 mM NaCl.

## Discussion

Salinity is one of the abiotic stresses that exert the greatest effects on agriculture worldwide [11]. One of the first processes affected by this stress in plants is photosynthesis and cell growth [32], which culminate in reductions in shoot and root growth. Studies using different species have revealed that the development of shoots is limited in plants that are susceptible to abiotic stress, whereas the growth of plants that are considered tolerant remains essentially unchanged [33,34]. In the present work, we used micropropagated shoots of two sugarcane cultivars (CB38-22 and RB855536) exposed to salt stress, and our results showed that the RB855536 cultivar had greater salt tolerance than the CB38-22 cultivar (Fig 1). As quoted in the methodology, farmers consider the CB38-22 cultivar as tolerant to salt, which is different from our experimental results. Although CB38-22 is an important cultivar planted in the region of Campos dos Goytacazes-RJ, this study describes the first assay of these cultivars under salt stress. Additionally, the micropropagated shoots used in our work were devoid of root systems and thus showed a faster response, and the effects were observed directly on leaf tissues. A more developed root system can lead to a greater tolerance to water stress and consequently to salt stress because the plant uptake water from deeper soil layers [35]. In this study, the use of an *in vitro* system was important for maintaining the controlled experimental conditions, which is required to associate the effects observed in the analysis with the induced salt stress response.

Two factors are very important in studies of abiotic stresses, particularly salt stress: the age of the plant and the concentration used to promote a stress condition. According to Shavrukov [36], the salt concentration used to promote salt stress is directly related to the type of damage caused by this stress in the plant, which ranges from osmotic stress under low concentrations to osmotic shock and plasmolysis under high concentrations. Recent studies have shown that the strategy of using juvenile plants and an NaCl concentration ranging from 100 to 200 mM is efficient for studying the molecular pathways associated with the response of sugarcane cultivars to salt stress [37,38]. Using this concentration range as a basis, the present study used 180 mM that was selected using a previously obtained curve (S1 Fig).

Long-term environmental changes, such as increased soil salinity, induce changes in different biochemical pathways. Salt stress can regulate the expression of numerous genes, resulting in significant changes in the cell proteome, including alterations in the expression of proteins directly involved in the plant’s response to stress [16]. In the present study, changes in the proteome were identified through the use of gel-free techniques followed by the label-free identification and quantification of proteins. This analysis allowed thorough protein identification and comparisons among the cultivars are important to determine the genotype-dependent responses and identify potential proteins related to the responses of sugarcane to salt stress. In particular, the greater abundance of proteins related to photosynthesis and ionic and oxidative homeostasis detected in the RB855536 cultivar compared with the CB38-22 cultivar can be directly correlated with the superior development and survival of RB855536 in the presence of salt stress.

An analysis of changes in the protein composition under stress and non-stress conditions is fundamental for understanding the major biochemical pathways involved in the molecular responses of plants [17]. The identification and functional analysis of key proteins related to these responses are important in the search for biomarkers [39]. We identified three proteins in both cultivars only during salt stress (S2 Fig and S1 Table), and two of these three proteins, hexokinase and GST, have been associated with responses to different abiotic stresses [40,41]. Because these proteins were observed in both cultivars only under salt stress conditions, they might be important proteins involved in the response to salt stress and might be useful as biomarkers associated with the response of sugarcane to salt stress.

In addition, in this study, we observed other groups of proteins that were regulated under stress conditions and might therefore play important role in the response to salt stress. The increase in the abundance of proteins related to photosynthesis is directly associated with increased plant growth under stress conditions (Fig 2). Proteins such as photosystem I subunit I (Sb03g004560.1), which exhibited a higher abundance in the stress-tolerant cultivar RB855536 than in CB38-22 (Fig 4 and Table 1), might be directly associated with a more robust response to salt stress conditions. An increase in the abundance of proteins associated with the photosystems is important for the prevention of severe damage to the photosynthetic apparatus and for the maintenance of energy production and plant development [42]. Maintaining a high photosynthetic rate under salt stress is a characteristic associated with tolerant sugarcane genotypes, which suggests that this factor is important for growth and development under these conditions [21]. Carbonic anhydrase-like 2 (γCAL2; Sb04g020630.1), which was discovered in *Arabidopsis thaliana* [43], is another photosynthesis-related protein that showed increased abundance under salt stress and might therefore play an important role in the response to salt stress. This protein forms part of complex I of the respiratory chain in the mitochondria of photosynthetic organisms and is an important enzyme in the carbon metabolism that catalyses the interconversion of CO_2_ and HCO_3_ [44]. The excess CO_2_ derived from the TCA cycle and photorespiration in the mitochondria becomes a substrate for γCAL2 and is converted to HCO_3_, which is transported to chloroplasts. In chloroplasts, HCO_3_ is converted into CO_2_, which servers as a substrate for ribulose-1,5-bisphosphate carboxylase/oxygenase (Rubisco) [45]. Therefore, an increase in γCAL2 might be important for reusing the CO_2_ produced in mitochondria through other biochemical pathways [46], and this protein is essential for regulating the carbon metabolism and for reducing the level of intracellular CO_2_, which might otherwise cause severe oxidative damage to the cell. An association between γCAL2 and an increased abundance of Rubisco and other photosynthetic proteins was also observed in the tolerant cultivar RB855536 (S1 Table). Therefore, this signalling pathway is associated with the regulation and greater efficiency of the photosynthetic apparatus and might play an important role in the response of sugarcane to salt stress.

Tolerant genotypes generally exhibit high photosynthetic rates under both stress and non-stress conditions. However, the photosynthetic rate is usually decreased in susceptible plants cultivated under abiotic stress [47]. This decrease in photosynthetic activity under stress conditions might affect energy metabolism and increase the production of ROS [7]. ROS are important signalling molecules during plant development; however, their overproduction during abiotic stress promotes intracellular accumulation and leads to the induction of oxidative damage [48]. Thus, the decreased abundance of the photosynthetic proteins Sb01g015400.1 and Sb03g020160.1 in the CB38-22 cultivar compared with the cultivar RB855536 (Table 1 and Fig 2) might induce ROS accumulation and consequently oxidative stress, preventing adequate growth of the susceptible cultivar. We found that a greater number of stress-response proteins, such as antioxidant proteins, were more abundant in CB38-22 (Figs 2 and 3), indicating that these might be directly associated with increased oxidative stress in this cultivar. Proteins such as peroxidase and GST act as ROS scavengers, preventing the intracellular accumulation of ROS [49]. Increased antioxidant enzyme activity is considered a marker of oxidative stress in a cell [50]. However, high expression of these proteins might not always be beneficial for improving tolerance to salt stress [51]. Thus, the effective participation of antioxidant enzymes might be species-dependent, and other antioxidant mechanisms might be more important and more efficient. Non-enzymatic antioxidants, such as flavonoids and osmolytes, might also be important in the oxidative stress response [52,53]. Plant genotypes, particularly sugarcane that exhibit tolerance to salt stress showed greater synthesis of secondary metabolites such as anthocyanins and flavonoids. These compounds can act directly in the response to stress, which suggests that these metabolites constitute an important response of sugarcane to salt stress [54].

Photosynthetic and antioxidant proteins are known to be tightly regulated under abiotic stress conditions. In this study, an examination of the differences in protein abundances between different cultivars revealed other classes of proteins that might be involved in the response of sugarcane to salt stress. The enzyme GAPC (Sb09g001600.1), which was found to be more abundant in the tolerant cultivar RB855536 under salt stress (RB-180 treatment; Fig 4 and Table 1), also plays a direct role in the cellular antioxidant mechanism. This enzyme is mainly known for participating in glycolysis, specifically for catalysing the conversion of glyceraldehyde 3-phosphate into 1,3-bisphosphoglycerate. However, it has other important functions in higher plants [55]. Under salt stress, GAPC plays a direct role in photosystem repair and consequently increases the photosynthetic rate. The acceleration of photosystem repair is achieved primarily by decreasing ROS accumulation and increasing CO_2_ fixation [56]. GAPC overexpression in *Oryza sativa* under salt stress effectively decreases the intracellular H_2_O_2_ concentration and consequently promotes greater tolerance [57]. In addition, *GAPC*-deficient mutants exhibit a lower respiration rate and decreased ATP production [58]. This decreased ATP production affects the energy metabolism of cells, which is driven by H^+^-ATPases that use ATP as a substrate. Thus, the increased abundance of GAPC in RB855536 compared with CB38-22 (Fig 4 and Table 1) might be directly related to the increased abundance and activity of H^+^-ATPase under salt stress in the more tolerant cultivar RB855536. H^+^-ATPase activity is an important response to salt stress, particularly due to the compartmentalization and ion exclusion of Na^+^ [11]. These processes are performed by an Na^+^/H^+^ antiporter that depends exclusively on the changes in the electrochemical gradient generated by H^+^-ATPases and H^+^-PPases [59]. The elevated expression of ion transport-related genes is important for increasing salt tolerance in sugarcane [60]. Thus, ion homeostasis together with oxidative homeostasis, which are regulated by the actions of the above-discussed proteins, might be essential for increasing the tolerance of sugarcane to salt stress.

In association with the increased production of ATP, the elevated activity of H^+^-ATPases are important for ionic homeostasis. In this context, calcium (Ca^2+^) serves as an important second messenger that activates H^+^-ATPases. The high concentration of intracellular Na^+^ induced by salt stress results in the accumulation of Ca^2+^ in the cytosol [61]. Errabii et al. [62] showed that susceptible sugarcane genotypes have a lower concentration of cytosolic Ca^2+^ than genotypes that are tolerant to salt stress. These researchers suggest that the difference in the cytosolic Ca^2+^ concentration between genotypes can be an important factor associated with the stress response. A high cytosolic Ca^2+^ concentration activates signalling cascades mediated by various proteins, including CDPK, which regulates the intracellular concentration of Na^+^, mainly by activating H^+^-ATPases [63]. Additionally, CDPKs are involved in other important signalling pathways that are activated in response to various abiotic stresses. These pathways include gene regulation, the absorption of potassium (K^+^), the scavenging of ROS, and the production of osmolytes [64]. In particular, CDPKs act synergistically with abscisic acid (ABA) and might be important for preventing tissue dehydration upon exposure to stress conditions. Yu et al. [65] revealed that this relationship between CDPK1 and ABA signalling is mainly related to the regulation of stomatal movement. In addition, CDPKs also regulate the expression of other proteins such as aquaporins, which are essential for the avoidance of excessive water loss [66]. Because osmotic stress is one of the main effects of severe salt stress, the higher abundance of CDPKs in the RB855536 cultivar compared with CB38-22 (Fig 4 and Table 1) might be directly associated with the higher tolerance of RB855536 to salt stress, which might be primarily associated with ionic and osmotic homeostasis under these conditions. Previous studies have shown that the overexpression of CDPKs increases the tolerance of several plant species to salt stress, suggesting the importance of these proteins in the response to salt stress [67,68]. In this context, our results suggest that CDPKs are important players in the response of sugarcane to salt stress conditions.

As previously mentioned, an increase in the concentration of cytosolic Ca^2+^, which acts as a second messenger, was observed during salt stress. Along with the aforementioned proteins, Ca^2+^ activates other important proteins, particularly phospholipases, in response to salt stress [69]. Among these proteins, PLD catalyses the rapid formation of lipid species, such as phosphatidic acid (PA), during exposure to different abiotic stresses [70]. PA plays roles in various stress signalling pathways [71]. In particular, this compound acts synergistically with ABA and CDPK in the regulation of important events such as stomatal closure and the activation of proton pumps [72,73]. Additionally, PLD can function in the structural re-organization of the plasma membrane [74]. Although it is strongly associated with the response to biotic stresses, this re-organization might also be important in the response to salt stress [70]. A recent study showed that PLD overexpression increases the tolerance of *A*. *thaliana* to salt and drought stresses [73]. These studies indicate the importance of PLD in the responses of multiple species to salt stress. In addition, phospholipases are important in the responses to other abiotic stresses, such as low temperature and drought [75]. Thus, the greater abundance of this protein in the RB855536 cultivar (Fig 4 and Table 1), which is the more tolerant cultivar, indicates that this protein might also be important in the response of sugarcane to salt stress.

In addition to the above-discussed proteins, the role of pathogenesis-related (PR) proteins in the response to abiotic stress remains unclear. Recent studies have demonstrated the potential involvement of PR proteins in defence mechanisms related to salt stress [76]. These proteins are considered important in the innate immune response of plants due to their involvement in responses to both pathogen attack and abiotic stimuli. PR proteins have been found to be more abundant under salt stress, and the overexpression of these proteins promotes an increase in salt tolerance [77]. This increase is primarily associated with signalling molecules that are important for defence mechanisms, such as methyl jasmonate, ABA, and salicylic acid [78,79]. In our studies, an increase in PR protein abundance was induced in the RB855536, and this effect might be directly associated with important hormone signalling pathways, such as the ABA, jasmonic acid, and ethylene pathways. Therefore, PR proteins might be important players in the response of sugarcane to salt stress (S1 Table).

Additionally, proteins involved in transcription and translation might also be important in the response to salt stress. Previous studies have shown that under stress conditions, epigenetic changes (i.e., DNA methylation and histone modifications) can regulate the expression of important genes related to stress tolerance [80] and can activate the transcription of genes that respond to abiotic stress [81]. Proteins involved in the regulation of transcriptional and translational activity are important because oxidative stress caused by high NaCl concentrations might affect protein integrity; thus, the synthesis of new proteins is critical for the maintenance of growth [82]. The abundance of eIF2 increases during salt stress, and this protein might be important in the synthesis of new proteins during the stress response [83]. eIF2 is responsible for the binding of RNA to the ribosomal subunit to initiate protein translation; therefore, it is a key protein in protein synthesis [84]. Pacheco et al. [21] showed that tolerant sugarcane genotypes present a greater abundance of proteins involved in protein biosynthesis, suggesting that these proteins are important in the response of sugarcane to abiotic stress. We found that the eIF2 protein level was higher in the tolerant cultivar RB855536 than in CB38-22 (Fig 4 and Table 1), indicating that this protein might also be important in the response of sugarcane plants to salt stress conditions. Therefore, proteins known to be involved in protein synthesis that show differential abundance during salt stress might be crucial for the synthesis of proteins in response to stress, the activation of signalling cascades, and the maintenance of plant growth and development under stress conditions.

## Conclusions

**Fig 5:**
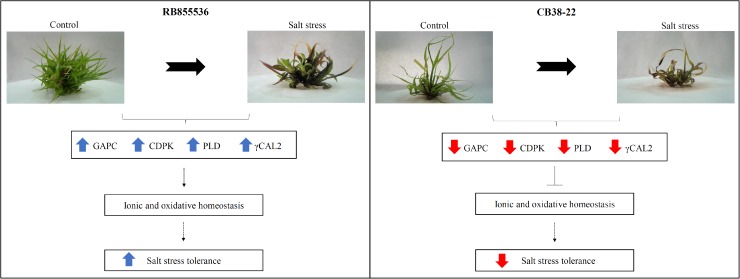
Proposed scheme of the major regulated proteins affecting ionic and osmotic homeostasis and thereby yielding a greater tolerance to salt stress in sugarcane. The blue arrows represent the more abundant proteins and red arrows represent the less abundant proteins when both cultivars were compared under salt stress.

The cultivar RB855536 was found to exhibit higher tolerance to salt stress than CB38-22, and proteins that presented differential abundance between these two cultivars might be directly associated with the altered tolerance and the maintenance of plant growth under salt stress. Proteins involved in non-enzymatic antioxidant mechanisms, ion transport, and photosynthesis were more abundant in the more tolerant cultivar, RB855536. The results suggest that the greater abundance of different groups of proteins may be directly associated with ionic and osmotic homeostasis in sugarcane during salt stress (Fig 5). In particular, the greater abundance of CDPK and PLD, which act directly in maintaining the normal concentration of Na^+^ in the cytoplasm and a lower accumulation of ROS, reduce the ionic and oxidative stress caused by salt stress. The proteins identified in the present study and their associated biochemical pathways provide new information regarding the tolerance of sugarcane to salt stress. Particularly in view of the lack of knowledge regarding these molecular responses in sugarcane, this study might aid the understanding of the physiological and molecular mechanisms used by cultivars in response to salt. Future performance analyses in the field will be important for confirming the response mechanisms employed by the cultivar RB855536.

## Supporting information

S1 Table Complete list of identified proteins. (XLSX)

S1 Fig Growth curve of the sugarcane cultivar RB855536 in response to different concentrations of NaCl. Shoot length (A), fresh matter (B), and dry matter (C) after exposure to 0, 75, 100, 150, and 180 mM NaCl for 15 days. The bars represent the means ± SD (n = 7). The same letter above the bars indicates no significant difference according to Tukey’s test at 5% probability.
(TIF)

S2 Fig Venn diagram illustrating the numbers of proteins identified under all of the conditions. The diagram shows unique proteins and proteins presenting differential abundances in the sugarcane cultivars CB38-22 and RB855536 after 20 days of incubation in MS culture media with 0 mM NaCl (control) or 180 mM NaCl. CB-0 and RB-0 = cultivars CB38-22 and RB855536, respectively, cultured without NaCl (controls); CB-180 and RB-180 = cultivars CB38-22 and RB855536, respectively, cultured with 180 mM NaCl.
(TIF)
