# Supplementary figures and images for: Salt stress induces changes in the proteomic profile of micropropagated sugarcane shoots
Source: PLoS One. 2017 Apr 18;12(4):e0176076. doi: 10.1371/journal.pone.0176076 (PMC5395195; doi:10.1371/journal.pone.0176076)

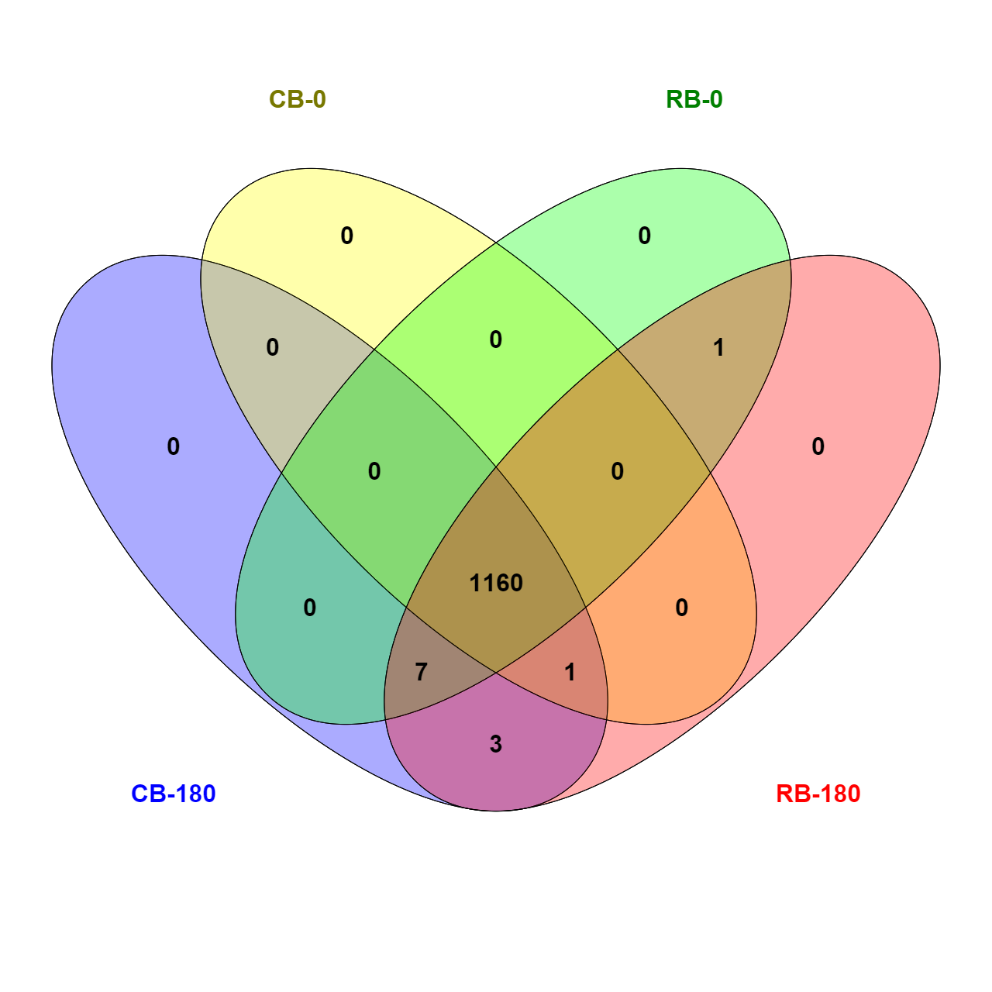

Supplement: S2 Fig — The diagram shows unique proteins and proteins presenting differential abundances in the sugarcane cultivars CB38-22 and RB855536 after 20 days of incubation in MS culture media with 0 mM NaCl (control) or 180 mM NaCl. CB-0 and RB-0 = cultivars CB38-22 and RB855536, respectively, cultured without NaCl (controls); CB-180 and RB-180 = cultivars CB38-22 and RB855536, respectively, cultured with 180 mM NaCl. (TIF) [file pone.0176076.s003.tif]
